# Types I and V Anti-CRISPR Proteins: From Phage Defense to Eukaryotic Synthetic Gene Circuits

**DOI:** 10.3389/fbioe.2020.575393

**Published:** 2020-09-30

**Authors:** Lifang Yu, Mario Andrea Marchisio

**Affiliations:** School of Pharmaceutical Science and Technology, Tianjin University, Tianjin, China

**Keywords:** CRISPR, anti-CRISPR, Cas proteins, synthetic biology, gene editing

## Abstract

Clustered regularly interspaced short palindromic repeats (CRISPR)-Cas (CRISPR-associated proteins), a prokaryotic RNA-mediated adaptive immune system, has been repurposed for gene editing and synthetic gene circuit construction both in bacterial and eukaryotic cells. In the last years, the emergence of the anti-CRISPR proteins (Acrs), which are natural OFF-switches for CRISPR-Cas, has provided a new means to control CRISPR-Cas activity and promoted a further development of CRISPR-Cas-based biotechnological toolkits. In this review, we focus on type I and type V-A anti-CRISPR proteins. We first narrate Acrs discovery and analyze their inhibitory mechanisms from a structural perspective. Then, we describe their applications in gene editing and transcription regulation. Finally, we discuss the potential future usage—and corresponding possible challenges—of these two kinds of anti-CRISPR proteins in eukaryotic synthetic gene circuits.

## Introduction

Clustered regularly interspaced short palindromic repeats (CRISPR)-Cas (CRISPR-associated proteins) is an RNA-mediated adaptive immune system that protects bacteria and archaea from being infected by bacteriophages and mobile genetic elements (MGEs). CRISPR-Cas contains two essential components: the CRISPR array and multiple *cas* genes (Barrangou et al., 2007; Garneau et al., 2010; Wiedenheft et al., 2012; Burstein et al., 2016). The CRISPR array consists of several non-continuous highly conserved DNA sequences, termed as *direct repeats*, that are separated by *spacers*. The latter represent DNA traits, of variable length and composition, that are homologous to those foreign elements (such as plasmids) that have previously entered the prokaryotic host cells. *Cas* genes are translated into multiple effector proteins that finalize the cleavage of the foreign nucleic acid aggressor (Bolotin et al., 2005; Pourcel et al., 2005; Barrangou et al., 2007). To date, CRISPR-Cas systems are classified, according to the effector Cas proteins, into 2 classes and six types. Each type is further divided into subtypes that show different architectures at the cleavage site on DNA or mRNA (Moon et al., 2019). Among the six types, type I, type III, and type IV belong to class 1, which requires the joint activity of multiple Cas proteins to induce the degradation of foreign DNA or RNA molecules. For example, CRISPR-Cas type I expresses the so-called Cascade—CRISPR-associated complex for antiviral defense (Zheng et al., 2020). In contrast, class 2, which gathers type II, type V, and type VI, involves a single multi-domain Cas protein, such as Cas9 (type II), Cas12a (previously called Cpf1—type V-A), and Cas13 (type VI) (Makarova et al., 2015; Koonin et al., 2017; Makarova et al., 2020).

The co-evolution theory claims that when an organism develops a new mechanism to defeat a parasite and avoid extinction, the parasite responds by creating proper countermeasures to circumvent the resistance of the host organism (Pawluk et al., 2018; Safari et al., 2020). Thus, bacteria avoided phage invasion by preventing phage adsorption (i.e., by blocking phage receptors activity), inhibiting phage DNA entry, and cleaving phage nucleic acids (e.g., via the R-M, restriction–modification, system). As a consequence, phages adopted new fighting strategies that involved the revision of the receptor-binding proteins and the removal, from their genome, of the recognition sites for the R-M system (Roberts et al., 2003; Labrie et al., 2010; Xu et al., 2010; Samson et al., 2013). The CRISPR-Cas systems in prokaryotic cells prompted tailored reactions from phages in order to re-establish the necessary conditions to replicate inside bacteria and archaea. Initially, phages achieved survival thanks to single nucleotide mutations or deletions in the conserved PAM or seed region along the protospacer (Deveau et al., 2008; Labrie et al., 2010; Semenova et al., 2011). However, with the increasing differentiation of CRISPR-Cas spacers inside bacterial populations, random point mutations and deletions were no longer adequate for phages to confront the CRISPR-Cas immune system (van Houte et al., 2016; Pawluk et al., 2018). In this context, phages further modified their genome to escape from being targeted by CRISPR-Cas immune system (Samson et al., 2013) and the anti-CRISPR proteins (Acrs) made their appearance (Pawluk et al., 2018). Anti-CRISPRs are small proteins (approximately, 12–193 amino acids) that confer to phages an efficient and powerful means to nullify the CRISPR-Cas immune system (Liu et al., 2020).

CRISPR-Cas systems have been largely used in gene editing and synthetic biology (Wright et al., 2016). Consequently, anti-CRISPR proteins have become, quickly, a new instrument to control CRISPR-Cas activity in biotechnological artifacts. This review aims at giving a detailed comparison of type I and type V-A anti-CRISPR proteins. We chose these two particular families since they act on CRISPR-Cas systems that belong to different classes and, hence, show remarkable dissimilarities in their working mechanism and overall complexity. The description of type I and type V-A CRISPR-Cas, in the next section, is preparatory to the AcrI and AcrV-A analysis that represents the core of this paper. In particular, we give a detailed picture of the structural changes and mechanisms at the basis of the working of these two types of anti-CRISPR proteins and we show how Acrs have been used, so far, for both *in vivo* and *in vitro* experiments. Finally, we discuss future potential applications of type I/V-A anti-CRISPRs in eukaryotic synthetic gene circuits and gene editing.

## Type I and Type V-A CRISPR-Cas Systems: General Features

Despite many differences, the six types of CRISPR-Cas systems show the same *action mode* in protecting prokaryotic cells. In order to cleave foreign nucleic acid molecules, every CRISPR-Cas system requires the formation of a complex containing an effector nuclease (e.g., Cascade or Cas12a) and a mature CRISPR RNA (crRNA), which comes from the transcription and processing of the CRISPR array.

CRISPR-based defense systems involve three distinct stages: adaptation, expression, and interference (Horvath and Barrangou, 2010; Barrangou and Marraffini, 2014). In the adaptation phase, Cas1—a metal-dependent integrase—forms a heterologous complex with Cas2, an adapter protein, to capture and process short stretches of foreign DNA/RNA sequences (the already mentioned spacers) upon recognition of the PAM (protospacer adjacent motif). Spacers are incorporated into the CRISPR locus at the promoter 5′UTR (untranslated region) (Yosef et al., 2012; Nunez et al., 2014; Lee et al., 2019; McGinn and Marraffini, 2019; Makarova et al., 2020). The protospacer adjacent motif plays a key role in self-/non-self-discrimination and, therefore, it is excluded from the CRISPR array (self-cleavage and auto-degradation would take place, otherwise) (Mojica et al., 2009). An additional nuclease protein, Cas4, is required in some subtypes such as type I-B, type II-B, and type V-A (Hudaiberdiev et al., 2017; Makarova et al., 2020).

During the expression phase, mature crRNAs are generated by endoribonucleases that process the precursor CRISPR RNA (pre-crRNA, i.e., the transcription of the CRISPR array). Different approaches are used by diverse CRISPR types and subtypes. In type I, from the archaeon *Pyrococcus furiosus*, Cas6 is the key enzyme to catalyze mature crRNA formation (Carte et al., 2008; Wang et al., 2011; Makarova et al., 2020). The same role as Cas6 is played by Csy4 in the bacterium *Pseudomonas aeruginosa* (Haurwitz et al., 2010; Wiedenheft et al., 2011). In type V, the subtype V-A demands a single Cas protein—Cas12a—to cleave the pre-crRNA and obtain the mature crRNA (Zetsche et al., 2015; Fonfara et al., 2016). However, in several type V subtypes such as type V-B, type V-E, and type V-F1—a branch of type V-F—pre-crRNA processing requires the presence of RNase III and *tracrRNA* (*trans*-activating crRNA) molecules (Sorek et al., 2013; Fonfara et al., 2014; Makarova et al., 2020), like in the well-known CRISPR-Cas9 system (Jinek et al., 2012; Faure et al., 2019; Makarova et al., 2020).

Finally, in the interference phase, the crRNA binds, by base pairing, the target nucleic acid in the proximity of PAM and puts the effector Cas protein in the condition to carry out DNA or RNA cleavage. As a consequence, the foreign DNA element is *inactivated*, i.e., quickly degraded (Makarova et al., 2020). At this stage, there are remarkable differences among the six CRISPR-Cas types.

The formation of Cascade in type I systems is considerably complex since it involves multiple Cas proteins such as Cas5, Cas7, Cas8, Cas6, and Cas11 (Sorek et al., 2013; Makarova et al., 2020). Furthermore, different type I subtypes follow distinct ways to assemble Cascade (Makarova et al., 2020). In contrast, type V requires a single Cas protein (Cas12) to form with crRNA the complex that cleaves the DNA (Sorek et al., 2013; Zetsche et al., 2015; Fonfara et al., 2016).

**FIGURE 1:**
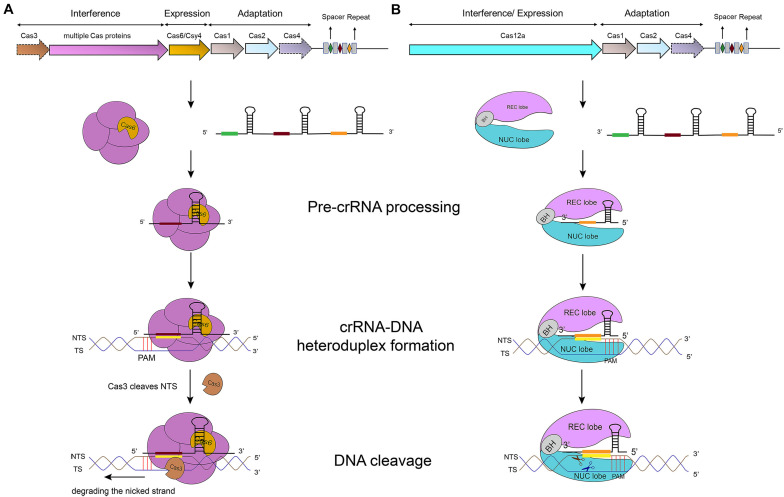
CRISPR-Cas-mediated pathways for DNA degradation. **(A)** Type I. Cas6 protein cleaves the pre-crRNA to generate the mature crRNA. Cascade-crRNA binds the DNA and recruits Cas3 that, first, induces a nick in the non-target strand (NTS), 7–11 nt downstream of PAM. Then, Cas3 degrades the nicked strand in an ATP-dependent manner. Afterward, Cas3 binds, cleaves, and degrades the target strand (TS) in the same way. Genes enclosed in a dotted line are not required by all subtypes. **(B)** Type V-A. Cas12a protein processes the pre-crRNA into mature crRNA and uses the only RuvC domain (in the NUC lobe) to cleave both strands of the substrate DNA, which provokes DNA degradation.

In type I systems, Cas3, an endonuclease that includes an N-terminal HD (histidine-aspartate) nuclease domain and a C-terminal superfamily 2 (SF2) helicase domain (DExD/H), is required to achieve ssDNA cleavage (Makarova et al., 2006). The Cascade-crRNA complex binds, with the help of PAM, the target strand (TS), which results in the formation of an R-loop to displace the non-targeted strand (NTS) of the substrate DNA (Rutkauskas et al., 2015). This R-loop functions as a loading space for Cas3. Upon Cas3 binding, the NTS is cleaved by the Cas3 HD nuclease domain—causing the formation of a nicked strand. Cas3, then, translocates along the NTS in the 3′–5′ direction by accepting energy from ATP hydrolysis carried out by its SF2 helicase domain. This movement provokes DNA unwinding and the NTS degradation. As a result, the target strand is exposed as an ssDNA. As such, also TS is bound, cleaved, and finally degraded by Cas3 (Westra et al., 2012) (see Figure 1A) (Sinkunas et al., 2013; Huo et al., 2014). In addition, in some type I subtypes (such as type I–F) Cas3 has to bind Cas2 in order to perform a catalytic cleavage (Westra et al., 2012; Huo et al., 2014; Makarova et al., 2020).

Cas12a presents several domains organized into two lobes: REC, the recognition lobe, that consists of REC1 and REC2 domains; and NUC, the nuclease lobe, that includes RuvC (cleavage), WED (wedge), PI (PAM-interacting), and Nuc domain. An arginine-rich bridge helix (BH) connects RuvC and REC giving rise to a bilobed architecture (Gao et al., 2016; Nishimasu et al., 2017). Between the lobes there is a central pocket—determined by REC lobe, RuvC, and WED—that hosts DNA molecules (Swarts et al., 2017). The Cas12a-crRNA complex leads to foreign DNA degradation in three steps. Initially, Cas12a-crRNA targets PAM to carry out DNA unwinding. In particular, some amino acids of PI and WED domain interact with PAM by forming hydrogen bonds and through van der Waals forces (Gao et al., 2016; Yamano et al., 2016, 2017). crRNA binds, by base pairing, the seed motif (5 nt) along the unwound DNA and forms a crRNA-DNA heteroduplex (about 20-nt long) with a simultaneous Cas12a configuration rearrangement. At this point, the R-loop is constituted on the non-targeted strand (as shown in Table 1). The R-loop architecture triggers the formation of salt bridges between Arg883 and Arg887 on BH and Glu939 on the lid region—made of the residues 924–940 of Cas12a. This facilitates the transition of a catalytic residue (Glu925) from the *closed* to *open* state that is required to carry out DNA cleavage (Stella et al., 2018; Zhang H. et al., 2019). Cas12a gets catalytically activated and the RuvC endonuclease domain makes two cuts on the foreign DNA, 18 and 23 nucleotides downstream of PAM on the non-targeted and the target strand, respectively. This causes a 5-nt staggered end away from the PAM sequence (Zetsche et al., 2015; Yamano et al., 2016) that induces DNA degradation (Gao et al., 2016; Swarts et al., 2017; Yamano et al., 2017; Zhang H. et al., 2019) (see Table 1 and Figure 1B).

**TABLE 1:** Features of type I and type V-A CRISPR-Cas systems.

Types	Type I	Type V-A
Size of the effector	From 300 to 450 amino acids	∼1,300 amino acids
Effector Cas proteins	Cas5/Cas7/Cas8/Cas6/Cas11	Cas12a
Pre-crRNA processing proteins	Cas6/Csy4	Cas12a
PAM	Variable. Type I-E: 5′-CAT. Type I-C: 5′-GAA	T-rich PAM: 5′-TTTV (V stands for “NOT T”)
Seed region	First 8 nt at the 5′ end of the spacer	First 5 nt at the 5′ end of the spacer
crRNA structure		
	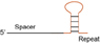	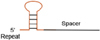
DNA-cleavage proteins	Cas3/Cas3-Cas2/Cas10d	Cas12a
Nuclease domain	HD	RuvC
Cleavage features		
	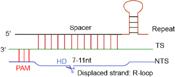	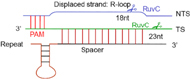

Different CRISPR-Cas types recognize diverse protospacer adjacent motifs. Cas12a (type V–A) recognizes *T-rich* PAMs—TTTV (where V stands for “NOT T”), whereas, in type I, PAM changes among subtypes. For instance, in type I-E, CAT is the most active PAM in *Escherichia coli* cells. However, in type I-C, GAA is the preferred PAM in *Bacillus halodurans* (Westra et al., 2013; Leenay et al., 2016; Lee et al., 2018, 2019).

Besides, DNA cleavage efficiency demands perfect complementary between the crRNA and the foreign DNA along the *seed* region, whereas mismatches are tolerated outside this region. In type V-A, the seed region corresponds to the first 5 bases at the spacer 5′ end (Zetsche et al., 2015). In type I, the seed sequence is longer (8 nucleotides) but still placed at the 5′ end of the spacer (Semenova et al., 2011; Wiedenheft et al., 2011) (see Table 1 for a schematic comparison of type I and type V-A CRISPR-Cas systems).

## Systems Classification

Type I CRISPR-Cas system includes 7 subtypes, labeled as type I-A up to type I-G (Makarova et al., 2015, 2020; Koonin et al., 2017). The kind and arrangement of multiple Cas proteins differ from subtype to subtype (Makarova et al., 2011, 2015, 2020). Cas proteins are divided into three groups. The Cas proteins that make the first group participate in the adaptation phase, i.e., the acquisition of foreign DNA. Cas1, a DNA-specific endonuclease (Wiedenheft et al., 2009; Babu et al., 2011; Yosef et al., 2012), and Cas2, which possesses both RNase and DNase activity [though not essential for adaptation (Beloglazova et al., 2008; Nam et al., 2012; Shah et al., 2013; Sorek et al., 2013; Nunez et al., 2014)], are core proteins present in all seven subtypes. Cas4 (Hooton and Connerton, 2014), the last protein in the first group, is absent from type I-E and type I-F (Makarova et al., 2015, 2020).

Cas proteins in the second group are the components of the Cascade effector. In type I-A, I-B, and I-C, only three *basic* proteins—Cas5, Cas7, and Cas8—are involved into Cascade composition, whereas type I-F, and I-G demand an extra protein, Cas6. Type I-E, together with Cas6, requires another protein, called Cse2 (Makarova et al., 2015). It should be noted that the Cas8 proteins in these seven subtypes share no significant sequence similarity (Makarova et al., 2020). In type I-D, the Cascade formation is different since Cas8 is replaced by Cas10d, which is a signature protein for type III (Makarova et al., 2011, 2015).

The third group coincides, mostly, with a single protein, Cas3, that has the capacity to unwind the crRNA-DNA heteroduplex and leads to DNA cleavage (Sinkunas et al., 2011; Gong et al., 2014; Huo et al., 2014). In type I-A and type I-D, the *cas3* gene presents two variants: *cas3*′, where the HD domain is fused to an SF2 helicase, and *cas3′′*, where, in contrast, the HD domain is not conjugated with other motifs (Beloglazova et al., 2011; Makarova et al., 2011). Type I-D shows a peculiarity also regarding group 3 Cas proteins since Cas3′′ is fused to Cas10d (Makarova et al., 2011, 2015, 2020).

In type V-A CRISPR-Cas system, the Cas effector is a single, multi-domain protein, initially termed Cpf1 and later renamed as Cas12a (Makarova et al., 2017). To date, 10 subtypes (the first nine indicated with the letters from A to I, and the last one with K) have been identified in type V (Makarova et al., 2020). Among all Cas12 proteins, Cas12a is the most studied and utilized in gene editing and synthetic gene circuits.

## Systems Applications

The CRISPR-Cas12a RNA-guided-endonuclease immune system drives fast and efficient gene editing (Hur et al., 2016) and achieves multiplex gene editing in the presence of a properly-designed CRISPR array (Tak et al., 2017; Zetsche et al., 2017; Campa et al., 2019). The DNase-deficient dCas12a protein is a template for the construction of new activators and repressors that are used, both in prokaryotes and eukaryotes, to wire the transcription units involved into synthetic gene circuits (Liu et al., 2017; Tang et al., 2017; Zhang et al., 2017). Differently from type V-A, type I CRISPR-Cas system has been rarely utilized in gene editing or synthetic transcriptional networks due to its complex architecture (Rath et al., 2015; Cameron et al., 2019; Pickar-Oliver et al., 2019).

Gene editing via CRISPR-Cas12a has some important limitations, though (Tu et al., 2017). One is the high number of off-target effects (Bondy-Denomy et al., 2013). Another is the small number of Cas12 orthologs that have been identified so far (Liu et al., 2017; Teng et al., 2019). However, recent results look promising. As shown in Teng et al. (2019), type V-A editing efficiency is enhanced by optimizing the loop region formed by the repeat sequence in the crRNA. Moreover a novel Cas12a—CeCas12a from *Coprococcus eutactus*—was reported to recognize PAM sequences more stringently than the other Cas12as currently used, with a consequent considerable reduction in the rate of off-target editing (Teng et al., 2019; Chen et al., 2020).

## Anti-CRISPR Proteins: A Defense Strategy Created by Phages

To date, 30 distinct families of anti-CRISPR proteins have been identified for type I and type V-A systems (Liu et al., 2020). Thirteen of them manifested on CRISPR-Cas an inhibitory action that is carried out in two different ways: either by preventing the Cascade/Cas-crRNA complex from binding the substrate DNA or by blocking DNA cleavage after inactivating the Cas effector.

### Type I Anti-CRISPR Proteins (AcrI): Discovery and Working Mechanisms

Over half of the anti-CRISPR proteins so far reported in the literature belong to type I. Overall, there are 25 AcrI families (Hwang and Maxwell, 2019; Zhang F. et al., 2019; Liu et al., 2020): 14 from subtype F (AcrIF1-F14), 7 from subtype E (AcrIE1-E7), and one apiece from subtype B, C and D. Moreover, the chimeric AcrIE4-F7 shows a dual type inhibition combining those of type I-E and type I-F. It is worth mentioning that AcrIF6 inhibits CRISPR-Cas type I-E as well. However, differently from AcrIE4-F7, the protein domains that interact with the two CRISPR-Cas type I subtypes are independent of each other and mutations in one domain do not affect the anti-CRISPR activity of the other domain (Pawluk et al., 2016b). The region that targets type I-F CRISPR-Cas is a motif conserved among several AcrIF6 homologs, whereas the anti-CRISPR type I-E functionality arises from some particular residues at the AcrIF6 C terminus (Pawluk et al., 2016b).

In 2013, the first anti-CRISPR protein was discovered in a prophage sequence inserted into the genome of *P. aeruginosa*. The prophage was supposed to be unable to replicate due to the presence of type I-F CRISPR-Cas. However, results from plaque assay were equivalent to those of PA14 ΔCR/cas (i.e., a *P. aeruginosa* strain without the CRISPR locus or *cas* genes), which pointed out that unknown proteins, encoded in the prophage, should have silenced the type I-F CRISPR-Cas activity. Comparative genomic analysis revealed that eight unique genes, located in a single locus of the genome of *P. aeruginosa* prophages, were responsible for the anti-CRISPR activity. Five of these genes were, then, associated with as many type I-F anti-CRISPR proteins (AcrIF1-F5) (Bondy-Denomy et al., 2013). Subsequently, four type I-E anti-CRISPR proteins (AcrIE1-E4) were discovered, again in *P. aeruginosa*, following a similar method (Pawluk et al., 2014). Sequences homologous to these nine type I anti-CRISPR genes were not found in other prokaryotic organisms. Furthermore, no particular motif is shared among them. The lack of conserved DNA traits prevented, initially, the uncovering of new anti-CRISPR proteins via bioinformatics methods. This impasse lasted until the Acr-associated (*aca*) genes, which encode for proteins with an HTH (helix-turn-helix) DNA binding domain, were spotted (Pawluk et al., 2016a,b; Marino et al., 2018). The first *aca* gene (*aca1*) was found downstream of each of the nine *acrI* genes in *P. aeruginosa*. Furthermore, a*ca1* was missing from phages lacking anti-CRISPR activity (Bondy-Denomy et al., 2013; Pawluk et al., 2014). By looking for Aca1 homologs with BLAST, four more type I-F anti-CRISPR proteins (AcrIF6-F10) were found in *P. aeruginosa* (Pawluk et al., 2016b). Later on, AcrIC1, AcrIE4-F7, AcrIE5-E7, and AcrF11-F14 were reported from other bacteria upon identification of the *aca1* gene (Marino et al., 2018). This approach was named “guilt-by-association.” Recently, *aca* genes were shown to work as repressors that control the activity of *acr*-associated promoters. The transcription of anti-CRISPR genes reaches high level immediately after the injection of the DNA of a phage into a prokaryotic cell. However, this transcription level is repressed in the presence of *aca* genes. In the absence of such a control, the strong transcription of *acr* genes would be lethal to phage (Stanley et al., 2019).

Even though many CRISPR-Cas systems occur in archaea, only two anti-CRISPR proteins were discovered from an archaeal virus, namely SIRV2 (*Sulfolobus islandicus* rod-shaped virus 2). The archaeon *S. islandicus*, which harbors a type I-D CRISPR-Cas system, could be infected by SIRV2 but not by a SIRV2 mutant lacking a 4-kb-long DNA fragment. Therefore, a type I-D anti-CRISPR activity was linked to genes in the lost 4 kb of DNA. Sequence comparison with the virus SIRV3 highlighted several conserved genes between the two viruses. Among them, a single gene from SIRV3, *gp02*, restored infectivity in the SIRV2 mutant, upon insertion into its genome. Hence, *gp02* was identified as the *acrID1* gene (He et al., 2018). The other anti-CRISPR protein of archaeal origin belongs to type III. This protein, termed AcrIIIB1, was identified in SIRV2 genome as well (*gp48* gene) (Bhoobalan-Chitty et al., 2019; Peng et al., 2020).

The latest AcrI protein, AcrIB1, was found recently in *Leptotrichia buccalis* DSM 1135 strains (Lin et al., 2020). The way it exerts its action, however, was not clarified yet.

So far, the CRISPR-Cas inhibitory mechanism of only 10 AcrI proteins (AcrID1, AcrIE1, AcrIF1-F4, AcrIF6, and AcrIF8-F10) has been understood. The way AcrIF1-F3, AcrIF6, and AcrIF8-F10 work was determined from their three-dimensional structures, whereas for the other three biochemical experiments were necessary. Eight AcrIs (AcrID1, AcrIF1-2, AcrIF4, AcrIF6, and AcrIF8-10) inactivate the CRISPR-Cas system by preventing Cascade from binding the target DNA (Bondy-Denomy et al., 2013, 2015; van Erp et al., 2015; Pawluk et al., 2016b; Chowdhury et al., 2017; Guo et al., 2017; Peng et al., 2017; He et al., 2018), whereas the remaining AcrIF3 and AcrIE1 disrupt the activity of the effector Cas3 that is involved in the interference phase (van Erp et al., 2015; Wang J. et al., 2016; Wang X. et al., 2016; Pawluk et al., 2017).

### Preventing DNA Binding

Type I-D CRISPR-Cas system is an unusual subtype since it appears to be a combination of type I and type III. Type I-D is characterized by the presence of both a Cas3 and a Cas10 variant. The former, termed Cas3′, belongs to type I (Jaubert et al., 2013), whereas the latter (Cas10d) should be a hallmark of type III (Niewoehner et al., 2017; Makarova et al., 2020). Unlike other type I subtypes, where Cas3 is responsible for DNA cleavage (Makarova et al., 2020), in type I-D it is Cas10d that cuts DNA. This rather big protein (∼90 kDa), indeed, contains an HD nuclease domain, which is usually found on Cas3. As mentioned above, AcrID1 was the first anti-CRISPR protein detected in archaeal viruses (He et al., 2018). AcrID1, probably, forms a dimer and binds two Cas10d molecules. As a result, Cas10d is no longer able to bind the target DNA (He et al., 2018; Makarova et al., 2020).

**FIGURE 2:**
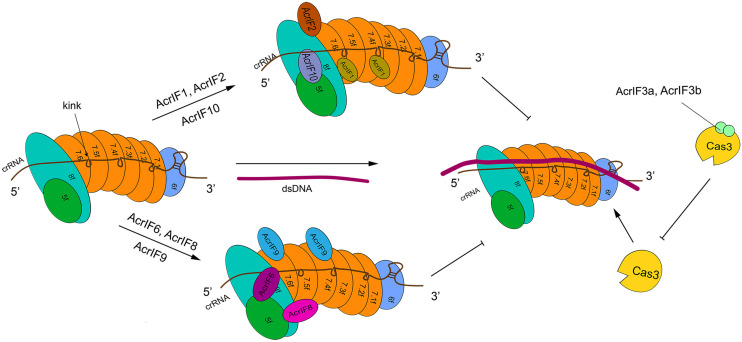
The structure of type I-F system and its interactions with AcrIF proteins. In the absence of AcrI proteins, the Csy-crRNA complex (made of Cas6f, Cas7.1f-7.6f, Cas5f–Cas8f, and the crRNA) binds the target DNA sequence. The resulting crRNA-DNA heteroduplex recruits Cas3 that carries out DNA cleavage. AcrI proteins prevent DNA degradation in two ways. AcrIF1, AcrIF2, AcrIF6, and AcrIF8-F10 hinder the interaction of Cascade with the DNA by binding different subunits of the Csy complex. In contrast, AcrIF3 blocks the function of Cas3. The precise inhibitory mechanism of AcrIF4 is still unknown.

Type I-F is characterized by the presence of a crRNA-guide surveillance (or Csy) complex that contains 4 critical Cas proteins (the *head*, Cas6f; the *backbone*, Cas7f, which is made of 6 subunits, from Cas7.1f to Cas7.6f; and the *tail*, which consists of two different proteins: Cas5f and Cas8f) and a crRNA complementary to the protospacer of a foreign DNA (Chowdhury et al., 2017; Peng et al., 2017) (see Figure 2). The four kinds of Cas proteins are required for PAM recognition and crRNA-DNA heterodimer formation (Guo et al., 2017). In particular, Cas6f first recognizes and cuts the crRNA stem-loop. In this way, a hairpin structure, to which Cas6f eventually binds, is formed at the crRNA 3′ end. Cas7f, which forms a helix along the crRNA, presents a “right hand” architecture composed by four domains: palm, main body, web loop, and thumb loop (Bondy-Denomy et al., 2015; Peng et al., 2017). The palm domain possesses a conserved RNA recognition region that responds to crRNA binding. The main body and the thumb loop make Cas7f form a highly symmetric helical structure. Moreover, these two domains generate a groove architecture for collecting crRNA (Peng et al., 2017). crRNA is accumulated on Cas7f protein via interactions that do not depend, specifically, on the actual crRNA sequence (Haurwitz et al., 2010; Wiedenheft et al., 2011; Chowdhury et al., 2017; Peng et al., 2017). Cas7f has a peculiar structure at the C terminus, called *extended web*, that is connected to both thumb and web domains—differently from what was reported for type I-E (Chowdhury et al., 2017; Peng et al., 2017). Cas7f folds together with the crRNA, which introduces a *kink* every 6 nucleotides along the crRNA. This corresponds to a 5 + 1 pattern (where the 6th nucleotide is buried such that a gap is formed) shared with other type I subtypes. This feature is possibly used to stabilize the crRNA-DNA heteroduplex together with the backbone (Taylor et al., 2015; Chowdhury et al., 2017; Guo et al., 2017; Peng et al., 2017). The tail of the Csy complex is formed by the Cas5f–Cas8f heterodimer and a conserved sequence in the last eight nucleotides of the crRNA 5′ end. This sequence gives rise to a S-shaped handle structure recognized by Cas5f–Cas8f (Chowdhury et al., 2017; Peng et al., 2017). Cas8f contains a *hook* domain at the N terminus followed by a central domain and a helical bundle at the C terminus. The latter interacts with Cas6f, Cas7f, and Cas5f (Bondy-Denomy et al., 2015; Chowdhury et al., 2017). Cas8f hook is essential in PAM recognition and crRNA-DNA heteroduplex formation since it generates a lysine-containing wedge (K-wedge) and an alanine-rich loop (A-loop) (Guo et al., 2017; Rollins et al., 2019). The K-wedge, indeed, reacts with the negatively charged DNA phosphatic backbone, which stabilizes the hybridization between crRNA and DNA. Moreover, the A-loop interacts with the second base of the PAM sequence. The crRNA-DNA hybridization leads to the formation of an R-loop in NTS. The R-loop permits the recruitment of Cas3 and a consequent DNA cleavage (Beloglazova et al., 2011; Mulepati and Bailey, 2011; Staals et al., 2016; Rollins et al., 2019).

AcrIF1 is a small protein made of only 78 amino acids. Three AcrIF1 residues (Y6, Y20, and E31) interact with a conserved lysine (K85) in the Cas7f thumb domain, which prevents crRNA-DNA hybridization (Bondy-Denomy et al., 2015; Maxwell et al., 2016; Chowdhury et al., 2017). The crystal structures of Csy-AcrIF1 and Csy-DNA permitted to understand that AcrIF1 binds both Cas7.4f and Cas7.6f in order to hinder DNA docking to the Csy complex. Furthermore, the polar interaction between Cas7f extended web and some residues on the C terminus of AcrIF1 is supposed to facilitate the adhesion of AcrIF1 to the Csy complex (Chowdhury et al., 2017).

AcrIF8 also interferes with crRNA-DNA hybridization but through a different mechanism. AcrIF8 protein (92 AA) locates into a pocket created by Cas5f, Cas7.4–7.6f, and Cas8f. From the Csy-AcrIF8 crystal structure, it is apparent that, because of a kink provoked by Cas7.5f thumb domain, three crRNA bases become so close to T29, I31, A32, and N33 AcrIF8 residues that both multiple hydrogen bonds and non-bonded interactions take place among them. The same kink interacts with AcrIF8 and causes the formation of a four-continuous-nucleotide region that removes the hybridization of crRNA with DNA and ends the crRNA-DNA heteroduplex propagation (Chowdhury et al., 2017; Zhang et al., 2020). Besides, the Csy-AcrIF8 complex might be stabilized by the interaction between residues of Cas7.5f (L94, K260) and AcrF8 (T43) (Zhang et al., 2020).

AcrIF9 action is distinct from that of AcrIF1 and AcrIF8, since AcrIF9 abolishes type I-F CRISPR-Cas function by competing with the substrate DNA for identical binding sites on the Csy complex. AcrIF9 is composed of 78 residues. Similar to AcrIF1, two molecules of AcrIF9 target Cas7.4f and Cas7.6f. AcrIF9 docks, because of electrostatic interactions, into a *vise-like* structure formed by positively charged lysines in the thumb and web loop region of Cas7f. The docking is further stabilized by other forces. AcrIF9 interacts, extensively, with lysine residues, of Cas7f subunits, that are responsible for DNA binding. Mutations of the residues of AcrIF9 that drive the interactions with Cas7f lysines result in direct DNA degradation, which means that AcrIF9 and DNA compete for lysines in Cas7f subunits (Zhang et al., 2020).

AcrIF6 (100 residues) targets the Cas5f—Cas8f tail and Cas7.6f via extensive non-bonded contacts. The Csy-AcrIF6 crystal structure revealed that two salt-bridges (D41:K247, D45:K247) and a hydrogen bond (Y38:T246) are formed between Cas8f and AcrIF6. Mutations of those AcrIF6 residues (D41A, D45A, 38A, or Y38W) that interact with K247 and T246 on Cas8f abolish the activities of AcrIF6 (Peng et al., 2017; Rollins et al., 2019; Zhang et al., 2020). Since K247 promotes DNA opening for crRNA-DNA hybridization and T246 converts DNA flipping into base-pairing with crRNA, AcrIF6 prevents the substrate DNA from binding the Csy complex by blocking the unwinding of the DNA duplex (Guo et al., 2017; Zhang et al., 2020).

AcrIF10 binds Cas5f—Cas8f tail stably, too. However, unlike AcrIF6, it works as a DNA mimic to inhibit the function of type I-F CRISPR-Cas system (Pawluk et al., 2016b; Guo et al., 2017). Cryo-EM structure of the Csy-AcrIF10 complex showed that AcrIF10 occupies a region of the Csy complex where some amino acids (e.g., Cas8f K77 and R78, Cas5f R90, and Cas7.6f K299) are involved in DNA binding. Hence, the interaction between the Csy complex—especially the Cas8f hook domain—and AcrIF10 decreases the probability of DNA binding. Moreover, the functionality of both K-wedge and A-loop in the hook domain, which facilitate the formation of the crRNA-DNA heteroduplex and PAM recognition, are weakened by AcrIF10 (Guo et al., 2017). Consistently with the AcrIF10 function as a DNA mimic, upon AcrIF10 adhesion, the Csy complex tail undergoes a structural change with the hook swinging toward Cas7.6f, which happens after DNA binding as well. However, differently from DNA binding, AcrIF10 binding to the Csy complex does not introduce a helical elongation and other structural changes along the backbone (Guo et al., 2017).

AcrIF2 works by mimicking the negative charge on the DNA. AcrIF2 wedges between Cas7.6f and Cas8f—without any interaction with Cas5f—and settles far away from Cas7.6f, which has no direct repercussion on substrate DNA binding to Cas7f backbone. The cryo-EM structure of Csy-AcrIF2 points out that a pseudo-helix is created by the acidic, negatively charged residues of AcrIF2. This motif acts on the positively charged residues in the hook and thumb region of Cas7.6f, which gives rise to a *lysine-rich*, *vise-like* structure. This particular structure precludes the binding of DNA to the Csy complex by breaking the interaction between the crRNA phosphate backbone and the Csy complex itself (van Erp et al., 2015; Chowdhury et al., 2017). Furthermore, the Csy-DNA cryo-EM structure shows that DNA binding makes Cas8f hook domain shift toward Cas7.6f subunit, whereas in the Csy-AcrIF2 configuration the hook domain swings away from Cas7.6f, which impedes the conformational changes necessary for substrate cleavage (Chowdhury et al., 2017; Guo et al., 2017; Peng et al., 2017; Rollins et al., 2019).

Another anti-CRISPR type I protein, AcrIF4, has been reported in the literature but its inhibitory mechanism is still unknown. Apparently, AcrIF4 binds Csy complex and inhibits the recruitment of Cas3 protein to the R-loop (Bondy-Denomy et al., 2015). No further information is, at present, available.

### Inactivating the Cas Effector DNase Activity

PaCas3 protein (from *P. aeruginosa*) is composed of an HD domain (i.e., a Ca^2+^-dependent nuclease domain), two RecA-like domains (RecA1 and RecA2 that form the SF2 helicase domain of PaCas3 and bind ATP), and a C-terminal domain (CTD). The connection of RecA2 and CTD is established by a long linker. The crystal structure of PaCas3-AcrIF3 reveals that PaCas3 HD domain is specifically bound by AcrIF3, whereas each RecA-like domain sticks to an ADP molecule. AcrIF3 works as a dimer, the two monomers being referred to as AcrIF3a and AcrIF3b. The interactions between AcrIF3 and the different PaCas3 domains drive the dimer to occupy the concave surface of PaCas3 due to the long linker and CTD (Bondy-Denomy et al., 2015; Wang X. et al., 2016).

Cas3 proteins from diverse type I CRISPR-Cas subtypes are, in general, considerably different. However, the substantial structure of type I-E TfCas3 (from *Thermobifida fusca*) and type I-F PaCas3 are highly similar (Huo et al., 2014; Wang X. et al., 2016). The CRISPR-Cas inhibitory mechanism put in place by AcrIF3 was clarified by comparing the structure of TfCas3-DNA and PaCas3-AcrIF3. AcrIF3, as a homodimer, has high affinity to PaCas3 and interacts with the RecA2 domain that shapes a *tunnel* with CTD for the substrate DNA binding—as it is apparent from the TfCas3-DNA structure. However, the tunnel formation is inhibited by AcrIF3 in a PaCas3-AcrIF3 complex, which decreases Cas3 accessibility to the target DNA (Huo et al., 2014; Wang X. et al., 2016).

In fact, in type I-E CRISPR-Cas, the Cse1 (also called CasA) subunit of Cascade—which is also responsible for PAM recognition (Sashital et al., 2012; Hochstrasser et al., 2014; Huo et al., 2014)—is required for recruitment of TfCas3 to the R-loop. During the interference stage, TfCas3 recognizes Cse1 via CTD and the long-linker region. However, in the PaCas3-AcrIF3 complex, AcrIF3 binds the concave surface of PaCas3, which prevents the usage of CTD and the long-linker region. As a consequence, Cse1 cannot be recognized, making PaCas3 fail to target the Csy-dsDNA complex (Wang X. et al., 2016).

ATP was reported to enhance Cas3 cleavage efficiency (Sinkunas et al., 2011; Mulepati and Bailey, 2013; Gong et al., 2014). Indeed, in the PaCas3-AcrIF3 complex, PaCas3 gets stuck in an ADP-bound form (Wang X. et al., 2016). Recently, Rollins et al. (2019) pointed out that AcrIF3 binds PaCas3 by mimicking the helical bundle in Cas8f, i.e., the domain bound by PaCas3. Upon DNA binding, the three-dimensional structure of the Csy complex changes and the helical bundle of Cas8f is rotated by 180°. This exposes a *nuclease recruitment helix* that has a structural homologous region on AcrIF3 (Rollins et al., 2019) (see Figure 2 for a schematic overview of type I-F anti-CRISPR protein working mechanisms).

The capability of AcrIE1 to bind Cas3 proteins and suppress their nuclease activity was ascertained by biochemical experiments. However, even though AcrIE1 structure has been obtained (Pawluk et al., 2017), the mechanisms through which AcrIE1 inhibits Cas3 is not fully understood yet.

### Type V-A Anti-CRISPR Proteins (AcrV)

So far, 10 subtypes of type V CRISPR-Cas system have been described (Altschul et al., 1997; Makarova et al., 2020). However, anti-CRISPR proteins (5 overall) were found only in subtype A (AcrVA1-A5). The discovery of AcrVA1-A3 was based on the presence of an *acrIF11* homolog in *Moraxella bovoculi* strains that contained CRISPR-Cas12a immune system and tolerated self-targeting sites in their genome (Marino et al., 2018). The identification of AcrVA4 and AcrVA5, also from *M. bovoculi*, was achieved through a new straightforward approach (Watters et al., 2018) that involved two technologies: STSS (self-targeting spacers search) and TXTL (cell-free transcription-translation) (Marshall et al., 2018).

AcrVA1 is a negatively charged protein that consists of 5 helices (α1–5) and binds a conserved region on Cas12a, which could explain why AcrVA1 is a broad inhibitor (Zhang H. et al., 2019). AcrVA1 blocks Cas12a-crRNA system in two ways. One is by mimicking PAM (Zhang H. et al., 2019). The cryo-EM map of the Cas12a-crRNA-AcrVA1 triple complex shows that AcrVA1 binds Cas12a central pocket tightly—via polar interactions since Cas12a is positively charged—and interacts with both NUC and REC lobe, especially with the WED domain. Loops, due to AcrVA1 helices, establish connections with WED and PI domain via residue interactions. They give rise to salt-bridges and hydrogen bonds such that some WED residues, which are responsible for interactions with PAM, get occupied by AcrVA1. This prevents the Cas12a-crRNA complex from interacting with PAM and carry out DNA cleavage (Zhang H. et al., 2019). The other operational mode of AcrVA1 provides for truncated crRNAs in a Cas12a-dependent fashion (Knott et al., 2019b; Suresh et al., 2019; Zhang H. et al., 2019). Mutations in key residues (D95A/S96A) of AcrVA1, which are responsible for Cas12a-crRNA-AcrVA1 complex formation, impair crRNA-cleavage. AcrVA1 α1 and α2 helices are positively charged. When AcrVA1 binds Cas12a-crRNA, crRNA is pinched by the two helices. Mutations in key residues of α2 helix restore Cas12a-mediated DNA cleavage efficiency (Suresh et al., 2019; Zhang H. et al., 2019). Therefore, α2 helix exerts RNase activity in crRNA-truncation (Suresh et al., 2019; Zhang H. et al., 2019).

AcrVA4 prevents DNA cleavage in more than a single way. AcrVA4 works as a dimer (Knott et al., 2019b) and binds two copies of the Cas12a-crRNA complex. In this way, Cas12a-crRNA ends up into a butterfly-shaped structure (Knott et al., 2019a,b; Zhang H. et al., 2019) that restrains the conformational changes that are required for the formation of the crRNA-DNA heteroduplex and the catalytic activation of Cas12a (Zhang H. et al., 2019). Moreover, the interaction between AcrVA4 and the Cas12a-crRNA complex is mediated by the β4–β5 and β6–α2 loops of AcrVA4 and the bridge helix of Cas12a (Zhang H. et al., 2019). The structure of Cas12a-crRNA-DNA shows that BH is involved in crRNA-DNA heteroduplex propagation and R-loop formation. The former demands polar interactions between BH and the crRNA-DNA heteroduplex, the latter a change in BH spatial conformation that is accomplished through a 180° rotation of the BH residue Arg887 (Swarts et al., 2017; Knott et al., 2019a; Zhang H. et al., 2019). This rotation is critical to establish a hydrogen-bond network between BH and the crRNA-DNA heteroduplex (Yamano et al., 2016, 2017; Zhang H. et al., 2019). In the Cas12a-crRNA-AcrVA4 complex, the β4–β5 loop forms a tight salt bridge with BH that locks the BH itself into the AcrVA4 structure. Hence, Arg887 rotation (and the corresponding conformational change in the crRNA-DNA heteroduplex formation) can no longer take place (Knott et al., 2019a; Peng et al., 2019; Zhang H. et al., 2019). Furthermore, the interaction between AcrVA4 β4–β5 loop and Cas12a REC lobe obstructs the REC2 movement necessary for crRNA-DNA heteroduplex propagation (Peng et al., 2019). BH is also involved in the transition of the lid region from the *closed* to the *open* state. When BH is locked in the interaction with AcrVA4, the lid region stays into a stable *closed* state that hinders DNA cleavage (Gao et al., 2016; Swarts et al., 2017; Stella et al., 2018; Zhang H. et al., 2019). AcrVA4 can also dislodge Cas12a-crRNA from the substrate DNA and, therefore, prevent target DNA cleavage by the CRISPR-Cas12a system. Only high concentrations of AcrVA4 make possible the release of the target DNA from the complex with Cas12a-crRNA (Knott et al., 2019b; Peng et al., 2019). Besides, AcrVA4 takes part in post-cleavage inhibition. In this case, AcrVA4 binds the Cas12a-crRNA-truncated-DNA complex and inactivate Cas12a activity, which decreases the recycling utilization of Cas12a (Peng et al., 2019). Finally, AcrVA4 binds Cas12a-crRNA also by mimicking a pre-crRNA substrate. This demands the recognition of both the 5′-hydroxyl group of mature crRNA and, above all, the pre-crRNA processing nuclease in the Cas12a WED domain. This behavior is significantly distinct from that of other Acr proteins that simply mimic DNA (Knott et al., 2019a). crRNA binding induces a rearrangement in Cas12a structure that provides the possibility for AcrVA4 to bind Cas12a-crRNA (Dong et al., 2016; Yamano et al., 2017; Knott et al., 2019a,b; Peng et al., 2019; Zhang H. et al., 2019). These results explain why AcrVA4 binding is crRNA dependent. Interestingly, AcrVA4 cannot inhibit AsCas12a (from *Acidaminococcus* sp. *BV3L6*) because of a helical insertion in AsCas12a that provokes a well-knit arrangement adjacent to the WED domain. This implies a steric clash with AcrVA4, which allows AsCas12a to escape from the AcrVA4-mediated inhibition (Knott et al., 2019a; Zhang H. et al., 2019).

**FIGURE 3:**
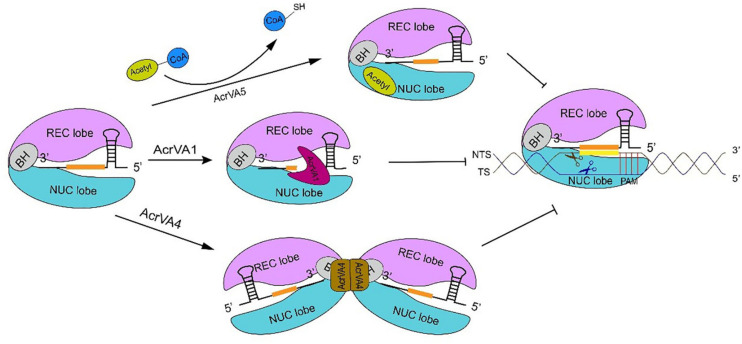
The inhibitory mechanism of AcrVA proteins. AcrVA1 and AcrVA4 prevent Cas12a-crRNA from binding dsDNA. AcrVA1 works by mimicking PAM to disrupt the communication among PI and WED (in Cas12a NUC lobe), and the DNA. Besides, AcrVA1 can also truncate the crRNA. In the complex Cas12a-crRNA-AcrVA4, AcrVA4 dimer drives two Cas12a-crRNAs to form a butterfly structure that prevents the structural change required for crRNA-DNA heteroduplex formation and catalytic cleavage activation. AcrVA5 is very distinct from the other two AcrVA proteins. In order to abolish Cas12a, AcrVA5 works as an acetyltransferase and transfers the acetyl group from acetyl-CoA to LbCas12a K595. Acetylated Cas12a can no longer interact with PAM. The sequence in orange represents the crRNA spacer.

AcrVA5 is very different from AcrVA1 and AcrVA4 since it works as an acetyltransferase and inactivates CRISPR-Cas12a systems by inducing covalent modifications. AcrVA5 inhibits both MbCas12a (from *M. bovoculi*) and LbCas12a (from *Lachnospiraceae bacterium ND2006*) by acting on a single residue—K635 (MbCas12a) or K595 (LbCas12a)—that is essential for interacting with PAM and unwinding the substrate DNA (Dong et al., 2019; Suresh et al., 2019). In LbCas12a-DNA crystal structure, K595 forms hydrogen bonds with TTTA PAM duplex via O2 in the third thymine and N3 in the complementary adenine of second thymine, whereas, the cryo-EM structure of LbCas12a acetylated by AcrVA5 shows that the acetylated-LbCas12a creates steric hindrance to PAM sequences and, hence, inhibits DNA binding (Yamano et al., 2017; Dong et al., 2019; Suresh et al., 2019). AsCas12a is insensitive to the activity of AcrVA5, even though K635 is conserved also in this CRISPR-associated protein (Dong et al., 2019) (see Figure 3 for a graphical comparison of the working of these three AcrVA proteins).

## A Summary of Anti-CRISPR Working Mechanisms

Type I and type V-A anti-CRISPR proteins carry out their action mainly by preventing Cascade/Cas12a-crRNA complex from recognizing and binding the target DNA or by abolishing DNA cleavage after inactivating the Cas effector. However, enzymatic behaviors have been reported too (Davidson et al., 2020; Wiegand et al., 2020).

With the exception of AcrIF3, subtype I-F anti-CRISPR proteins preclude direct reactions between Cascade-crRNA and the DNA via the suppression of the target DNA docking to the Csy complex. AcrIF1, AcrIF8, and AcrIF6 interact with residues, located on the backbone and the tail of the Csy complex, and interfere with the base-pairing between the crRNA and the target DNA. In contrast, AcrIF9 competes with the DNA for binding the Csy complex backbone at lysine residues, whereas AcrIF10 occupies a region of the Csy complex directly involved in DNA binding. AcrIF2 imitates DNA negative charge and wedges between Csy complex backbone and tail, which blocks any DNA binding—and also hinders conformational changes necessary for DNA substrate cleavage.

AcrIF3 mimics the helical bundle in the Csy complex tail where the effector Cas3 binds. Hence, Cas3 is no longer recruited by the Cascade-crRNA complex with the consequent impossibility to cleave DNA. An interaction with Cas3 is supposed to be at the basis of the working of AcrIE1 as well, whereas AcrIF4 probably impedes the recruitment of Cas3 protein to the R-loop. Finally, AcrID1 is supposed to form a dimer and bind two Cas10d molecules, which excludes any possible binding between Cascade-crRNA and the DNA.

As for type V-A anti-CRISPR proteins, AcrVA1 works by mimicking PAM, which disrupts the interactions between Cas12a-crRNA complex and the target DNA. Moreover, AcrVA1 exerts an enzymatic action by cutting crRNA molecules bound to Cas12a.

A different enzymatic behavior is manifested by AcrVA5. This anti-CRISPR protein is an acetyltransferase that inactivates CRISPR-Cas12a by inducing covalent modifications. AcrVA5 inhibits both MbCas12a and LbCas12a by acting on a single residue essential for both the interactions with the protospacer adjacent motif and DNA unwinding.

Like AcrVA1, AcrVA4 shows different action modalities. AcrVA4 works as a dimer and binds two copies of the Cas12a-crRNA complex, which hinders the conformational changes necessary to create the crRNA-DNA heteroduplex and activate Cas12a. Furthermore, AcrVA4 is able to dislodge Cas12a-crRNA from the substrate DNA. Finally, AcrVA4 can bind a Cas12a-crRNA-truncated-DNA complex and inactivate Cas12a (post-cleavage inhibition). As a result, Cas12a cannot be recycled for further DNA cutting (see Table 2).

**TABLE 2:** Type I and type V anti-CRISPR proteins.

Inhibition mode	Type	Name	Residues	Target	Inhibitory mechanism	Inhibited CRISPR-Cas type	Gene editing target	Transcription regulation target	Accession number	References
DNA binding	I	AcrID1	96	Cas10d	Possible allosteric inhibition by inducing Cascade-crRNA dimerization	Type I-D	–	–	YP_009272954.1	Cameron et al., 2019
		AcrIF1	78	Cas7.4f and Cas7.6f	Blocking crRNA-DNA hybridization	Type I-F	–	–	YP_007392342.1	Bondy-Denomy et al., 2015; Guo et al., 2017; Tang et al., 2017; Tu et al., 2017; Teng et al., 2019; Chen et al., 2020
		AcrIF2	90	Cas7.6f and Cas8f	Mimicking the negative charge on DNA and disrupting the interaction between the crRNA phosphate backbone and the Csy complex	Type I-F	–	–	NP_938237	Bondy-Denomy et al., 2015; Tang et al., 2017; Hwang and Maxwell, 2019; Teng et al., 2019; Chen et al., 2020
		AcrIF4	100	–	–	Type I-F	–	–	WP_016068584.1	Liu et al., 2017; Tu et al., 2017
		AcrIF6	100	Cas5f—Cas8f tail, and Cas7.6f	Interaction with Cas8f (at K247) that prevents DNA opening for crRNA-DNA hybridization	Type I-E/Type I-F	–	–	WP_043884810	van Erp et al., 2015; Liu et al., 2017
		AcrIF8	92	Cas5f, Cas7.4–7.6f, and Cas8f	Interaction with crRNA that disrupts crRNA hybridization with DNA and prevents crRNA-DNA heteroduplex propagation	Type I-F	–	–	AFC22483.1	van Erp et al., 2015; Liu et al., 2017
		AcrIF9	68	Cas7.4f and Cas7.6f	Competition with DNA for the lysines in Cas7f subunits that are responsible for DNA binding	Type I-F	–	–	WP_031500045.1	van Erp et al., 2015; Liu et al., 2017
		AcrIF10	97	Cas5f—Cas8f tail	Competition with DNA (via DNA mimic) for binding Cas5f-Cas8f	Type I-F	–	–	KEK29119	Liu et al., 2017; Chen et al., 2020
	V	AcrVA1	26	Central pocket of Cas12a	(1) Interaction with WED and PI domains, PAM sequence mimic. (2) Truncation of crRNA in a Cas12a-dependent way.	Type V-A	Human cells: Mb, Lb, As and FnCas12a Bacteria: MbCas12a	Mammalian cells: dLbCas12a-miniVPR	WP_046701302.1	Sashital et al., 2012; Mulepati and Bailey, 2013; Hochstrasser et al., 2014; Niewoehner et al., 2017; Zetsche et al., 2017
		AcrVA4	35	Helix bridge (HB), WED domain (pre-crRNA processing nuclease) and 5′-hydroxyl group of cr-RNA	(1) Inhibition of Cas12a conformational changes required for catalytic activity. (2) Dislodging Cas12a-crRNA from DNA. (3) Binding to Cas12a-crRNA-truncated-DNA complex to decrease the recycle of Cas12a.	Type V-A	Human cell: LbCas12a, MbCas12a	–	WP_046699156.1	Altschul et al., 1997; Sashital et al., 2012; Hochstrasser et al., 2014; Niewoehner et al., 2017; Watters et al., 2018
		AcrVA5	12	–	Permanent inactivation of Cas12a via covalent modification (acetyltransferase activity)	Type V-A	Human cell: LbCas12a, MbCas12a	–	WP_046699157.1	Sashital et al., 2012; Mulepati and Bailey, 2013; Niewoehner et al., 2017; Knott et al., 2019b
Effector inactivation	I	AcrIE1	100	Cas3	–	Type I-E	–	Bacteria: PaCas3	YP_007392738.1	Zhang et al., 2017; Zhang F. et al., 2019
		AcrIF3	139	Cas3	Binding Cas3 by mimicking the helical bundle of Cas8f.	Type I-F	–	Bacteria: PaCas3	YP_007392440.1	Wiedenheft et al., 2011; Tang et al., 2017; Tu et al., 2017; He et al., 2018
Unknown	I	AcrIB1	193	–		Type I-B	–		WP_015769207.1	Lin et al., 2020
		AcrIC1	180			Type I-C			AKG19229.1	Zetsche et al., 2017
		AcrIE2	84			Type I-E			YP_007392439.1	Zhang et al., 2017
		AcrIE3	68			Type I-E			YP_950454.1	Zhang et al., 2017
		AcrIE4	52			Type I-E			NP_938238.1	Zhang et al., 2017
		AcrIE5	65			Type I-E			WP_074973300.1	Zetsche et al., 2017
		AcrIE6	79			Type I-E			WP_087937214.1	Liu et al., 2017
		AcrIE7	106			Type I-E			WP_087937215.1	Liu et al., 2017
		AcrIE4-F7	119			Type I-E/Type I-F			WP_064584002.1	Zetsche et al., 2017
		AcrIF5	79	–		Type I-F	–		YP_007392740.1	Zhang et al., 2017
		AcrIF7	67			Type I-F			ACD38920.1	Liu et al., 2017
		AcrIF11	120			Type I-F			WP_038819808.1	Zetsche et al., 2017
		AcrIF12	118			Type I-F			ABR13388.1	Zetsche et al., 2017
		AcrIF13	110			Type I-F			EGE18854.1	Zetsche et al., 2017
		AcrIF14	125			Type I-F			AKI27193.1	Zetsche et al., 2017
	V	AcrVA2	36	–		Type V-A	Bacteria: MbCas12a	–	AKG19228.1	Zetsche et al., 2017
		AcrVA3	18			Type V-A			AKG19230.1	Zetsche et al., 2017

Beside the type I and type V anti-CRISPR proteins, twenty-six anti-CRISPRs that act on type II-A and type II-C CRISPR-Cas systems have been discovered. They show a variety of mechanisms to annihilate the CRISPR-Cas working. AcrIIA1 and AcrIIC2, for instance, prevent the formation of the Cas9-crRNA-tracrRNA complex. Both bind to Cas9: AcrIIA1 induces Cas9 degradation, whereas AcrIIC2 hinders the guide RNA loading. AcrIIA2 and AcrIIA4 mimic DNA and, as a consequence, occlude Cas9 PAM-recognition site. AcrIIC1 and AcrIIC2 bind Cas9 at the catalytic site of the HNH nuclease domain. In this way, the CRISPR-Cas9 complex can still bind the DNA but cannot cleave it. Other type II Acrs, such as AcrIIA6 and AcrIIC3, carry out their function as allosteric inhibitors, i.e., they induce conformation changes, which preclude CRISPR-Cas9 working, upon interaction with non-functional Cas9 sites. Finally, AcrAII5 was shown to behave as an enzyme and cleave the guide RNA at multiple places, outside the spacer sequence (Hardouin and Goulet, 2020).

Only two type III Acrs have been reported so far: AcrIII-1 (Athukoralage et al., 2020) and AcrIII-B1. Both proteins seem to degrade cyclic tetra-adenylate molecules that are produced by Cas10 in order to activate the Csx1 RNase. Due to the particular features of type III CRISPR-Cas system (Molina et al., 2020), Csx1 action is required to free prokaryotic cells from viral transcripts.

Type VI CRISPR-Cas systems target, only, the RNA derived by foreign DNA elements. Seven type VI Acrs have been discovered so far. They prevent RNA targeting either by binding the Cas13-crRNA complex (AcrVIA1-A3) or just Cas13 (AcrVIA1 and AcrVIA4-A6) (Meeske et al., 2020).

## The Applications of AcrI and AcrV Proteins in Transcriptional Control and Gene Editing

As we have mentioned above, CRISPR-Cas type I system is characterized by the presence of the Cascade complex that gathers multiple Cas proteins. Cascade composition is, however, not fixed but varies among type I subtypes and the precise location of the cleavage site in NTS is still unknown (Mulepati and Bailey, 2013; Hochstrasser et al., 2014; Huo et al., 2014). All these difficulties slowed down the development of gene editing tools and the construction of synthetic transcription factors based on the CRISPR-Cas type I system. Consistently, the anti-CRISPR type I proteins could be used rarely inside synthetic gene circuits, despite the fact that they were the first anti-CRIPSR proteins discovered in nature.

AcrIE1 and AcrIF3 abolish type I immune function by preventing Cas3 helicase-nuclease from being recruited by the Cascade-crRNA complex. Upon expression of AcrIE1 or AcrIF3, Cascade-crRNA binds the substrate DNA stably without triggering any DNA degradation. Therefore, AcrIE1 and AcrIF3 are a means to regulate gene transcription (Luo et al., 2015; Marino et al., 2020). In 2015, Rath et al. (2015) utilized the Csy complex as a repressor of GFP in *E. coli* and *Salmonella typhimurium*. Cas3 was absent from this circuit, whereas crRNAs were designed to target the promoter upstream of GFP or the fluorescence protein sequence itself. In the same year, Luo et al. (2015) and Marino et al. (2020) proved that, upon deletion of Cas3, type I-E CRISPR-Cas system can be converted into a programmable gene regulator to monitor the expression of heterologous and endogenous genes in *E. coli*. By following a similar approach, Bondy-Denomy et al. (2015) pointed out that AcrIF3 turns CRISPR-Cas type I-F system into a transcriptional repressor. In their work, AcrIF3 was employed to modulate the production of the blue–green pigment pyocyanin in *P. aeruginosa*. The crRNA was designed to bind (in association with the Csy complex) the *phzM* promoter located upstream of the pyocyanin gene. A slightly modified *P. aeruginosa* strain (Csy/ΔCas3) was used as a control. Strains expressing Csy-AcrIF3 generated an amount of pyocyanin similar to that of the control cells, i.e., both of them disrupted the generation of pyocyanin. In this work, Csy-AcrIF1/AcrIF2 systems were also shown to behave very differently from Csy-AcrIF3 and Csy alone, i.e., they did not exert any inhibition on pyocyanin production. This was due to the fact that AcrIF1 and AcrIF2 prevented the Csy complex from binding the *phzM* promoter, whereas AcrIF3 binds and inactivates Cas3 nuclease. The Csy complex, on its own, has access to the *phzM* promoter and represses transcription strongly. Pawluk et al. (2017) reported that AcrIE1 protein could be repurposed as a programmable transcriptional repressor in *P. aeruginosa* via an analogous approach. They designed two crRNAs targeting the -10 and the -35 sequences of *phzM* promoter. Besides, they used, as a positive control, a strain without a CRISPR-Cas system (ΔCRISPR-Cas) and, as a negative control, a strain containing an inactive Cas3 mutant (H89A/D90A). AcrIE1 led to a decrease in pyocyanin production—as in the negative control—compared to the positive control that showed normal pyocyanin production (Marino et al., 2020).

**FIGURE 4:**
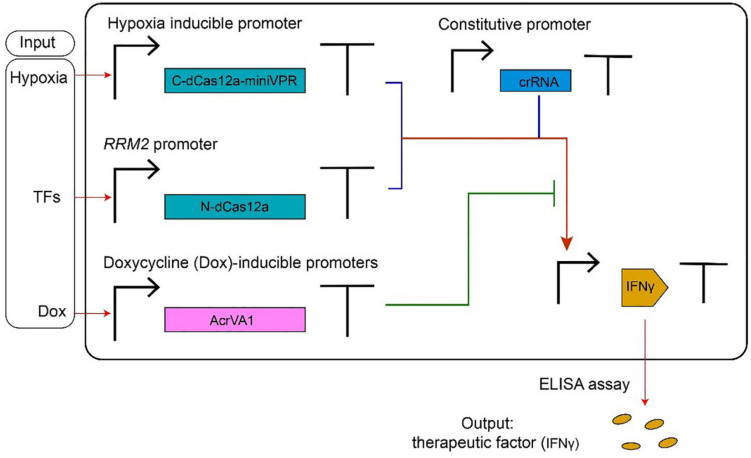
AcrVA1-mediated multiple-input synthetic gene circuit. The genetic network in the figure expresses the therapeutic factor IFNγ, in a controlled way, upon detection of tumor-relevant signals in HEK293T cells. The chimeric activator dCas12a-miniVPR-crRNA (where miniVPR is an activation domain) regulates the synthesis of IFNγ, the circuit output. dCas12a-miniVPR is split into two parts: the N-terminal dCas12a (N-dCas12a) and the C-terminal dCas12a fused to miniVPR (C-dCas12a-miniVPR). Both parts are expressed under inducible promoters: the hypoxia-inducible promoter (hypoxia can signal the presence of a tumor) leads C-dCas12a-miniVPR synthesis, whereas the endogenous *RRM2* promoter (ribonucleotide reductase regulatory subunit M2) drives the production of N-dCas12a in response to TFs (transcription factors) whose occurrence is due to a tumor. crRNA molecules, which bind dCas12a-miniVPR, are constitutively transcribed. AcrVA1 amount is controlled by the antibiotic doxycycline (Dox). When two tumor-relevant signals (hypoxia and TFs) appear in the cells, IFNγ are produced and can be detected by the ELISA assay. AcrVA1 is used to modulate the activity of dCas12a-miniVPR-crRNA and, hence, the level of the output signal.

Type V anti-CRISPR proteins have been adopted, mainly, in gene editing. Their usage in controlling transcription factors based on CRISPR-dCas12a systems has been shown in a recent paper by Kempton et al. (2020). Here, AcrVA1 mimics an OFF switch (NOT logic operation) inside a logic synthetic gene network (see Figure 4 for details).

Marino et al. (2018) reported that AcrVA1 blocked gene editing by Cas12a-crRNA in human U2-OS cells, whereas AcrVA2, AcrVA3, and AcrVA3.1—an ortholog of AcrVA3 with which it shows 43% of sequence identity—exhibited little evidence of activity against gene editing. Marino and co-authors chose, initially, an enhanced green fluorescent protein (*EGFP*) as a target, in order to evaluate AcrVA efficiency. Cells expressing AcrVA1 together with the MbCas12a-crRNA system returned a fluorescence signal much higher than that of the reference strain, where only MbCas12a-crRNA was present. In contrast, AcrVA2, AcrVA3, and AcrVA3.1 did not modify, drastically, the effects MbCas12a-crRNA on EGFP synthesis. Similar results were obtained by employing Mb3Cas12a instead of MbCas12a. AcrVA1 was, then, shown to inhibit Cas12a-crRNA disruptive action also on human endogenous genes such as *RUNX1*, *DNMT1*, and *FANCF*. Overall, AcrVA1 appeared to be a broad-spectrum anti-CRISPR protein in human cells, able to exert strong inhibition on MbCas12a and Mb3Cas12a, and a more modest suppression of LbCas12a, FnCas12a (from *Francisella novicida U112*), and AsCas12a activity. In a parallel work, Watters et al. (2018) confirmed the inhibitory action of AcrVA1 in human cells (here, embryonic kidney—HEK293T—cells were used). AcrVA1 was highly effective on AsCas12a, whereas less evident was its action on LbCas12a (data on MbCas12a was not collected since MbCas12a did not lead to any efficient gene editing). Beside AcrVA1, AcrVA4, and AcrVA5 were considered in this work too. Both AcrVA4 and AcrVA5 blocked LbCas12a-based gene editing but failed to inactivate AsCas12a. As mentioned above, Knott et al. (2019a) explained that a structural motif—an ancestral helical bundle—present on AsCas12a is what prevents AcrVA4 and AcrVA5 binding. Interestingly, two chimeric proteins, termed As^∗^Cas12a and Lb^∗^Cas12a, were engineered by swapping the helical bundle between AsCas12a and LbCas12a. In the absence of AcrVA4, both As^∗^Cas12a and Lb^∗^Cas12a led to proper gene editing in HEK293T cells. However, in the presence of AcrVA4, As^∗^Cas12a-mediated gene editing was completely abolished, whereas Lb^∗^Cas12a-crRNA was able to cleave the target DNA. This was the first work where the interaction between a Cas effector and an anti-CRISPR protein was drastically modified by re-engineering a Cas protein domain. As for bacterial cells, Marino et al. (2018) pointed out that AcrVA1 could interfere successfully with gene editing in *P. aeruginosa* strains that expressed MbCas12a-crRNA targeting DNA from *P. aeruginosa* phage JBD30. From phage plaque assay, it was evident that AcrVA1 restored phage replication robustly, namely, at the same level as the positive control circuit that had no crRNA for guiding MbCas12a to bind the target DNA. AcrVA2 (and one of its ortholog, termed AcrVA2.1) also inhibited MbCas12a-based gene editing in *P. aeruginosa* strains, whereas AcrVA3 manifested little inhibition and, above all, toxic effects to cell growth. AcrVA3.1 presented a stronger repression of MbCas12a-crRNA activity without toxicity. Interestingly, AcrVA3.1 proved to be partially efficient also against type I-C CRISPR-Cas system. Thus, it is considered to be a *dual-specific* inhibitor.

AcrVA1, AcrVA4, and AcrVA5 have been tested also *in vitro*. The results were similar to those *in vivo*: AcrVA1 inhibited all three Cas12a-crRNA (As-, Lb-, and MbCas12a) systems, with the strongest effect on MbCas12-crRNA and the weakest on AsCas12a-crRNA. As for AcrVA4 and AcrVA5: they blocked LbCas12a and MbCas12a efficiently but could not work on AsCas12a (Watters et al., 2018; Knott et al., 2019b) (see Table 2).

## Discussion

In type I CRISPR-Cas systems, the main binding sites for anti-CRISPR proteins are Cas7 (backbone) and Cas8 (tail). AcrIF1 and AcrIF8 target different subunits of Cas7f, whereas AcrIF10 binds Cas5f–Cas8f. AcrIs that bind identical Cascade subunits can share similar inhibitory mechanisms. In type I-F CRISPR-Cas, AcrI proteins located on Cas8f tail (e.g., AcrIF10 and AcrIF2) work by mimicking the DNA and preventing PAM recognition by the Cascade-crRNA complex. Anti-CRISPR proteins, such as AcrIF1 and AcrIF8, that bind Cas7f backbone—which plays an essential role in crRNA recruitment—possibly affect the crRNA-DNA hybridization and crRNA-DNA heteroduplex propagation. In type V CRISPR-Cas systems, AcrVA1, AcrVA4, and AcrVA5 employ different strategies to annihilate Cas12a-crRNA. AcrVA1 reminds of AcrIF10 since it competes with crRNA for the access to PAM. However, AcrVA1 also displays RNase activity that results in crRNA truncation. In contrast, AcrVA4 induces structural changes in Cas12a proteins in order to hinder DNA cleavage. AcrVA5, finally, works as an enzyme that wipes out permanently CRISPR-Cas12a nuclease activity through a covalent modification on Cas12a.

Some anti-CRISPR proteins show unique working mechanisms. For instance, AcrIF3, is the only protein that mimics a Cas protein (Cas8f) rather than a DNA sequence (Rollins et al., 2019). This indicates that prokaryotes and phages, through co-evolution, had to develop distant variants of their defending and invading systems, respectively. We think that this will result, in the future, in the discovery of novel CRISPR-Cas systems, and corresponding anti-CRISPR proteins, quite different from those so far encountered. AcrIE1 and AcrIF3 bind and inhibit the Cas3 DNase. However, it is not understood yet the way AcrIE1 works (Pawluk et al., 2017). Both AcrIE1 and AcrIF3 convert the CRISPR-Cas system into a transcriptional repressor by inactivating Cas3 protein (Bondy-Denomy et al., 2015; Pawluk et al., 2017). Thus, AcrIE1 and AcrIF3 might be employed inside synthetic transcriptional networks. AcrIF6, from *P. aeruginosa*, is a particularly interesting anti-CRISPR type I-F protein that can abolish, beside type I-F, also type I-E-mediated immune system. Two different protein domains are responsible for interacting with distinct CRISPR-Cas systems (Pawluk et al., 2016b). However, applications based on AcrIF6 are relatively limited. The special inhibitory mechanism of AcrIF6 might become a dual off-switch in complicated circuits that contain both type I-E and type I-F CRISPR-Cas systems and deal with multiple inputs.

Three natural type V-A anti-CRISPR proteins (AcrVA1, AcrVA4, and AcrVA5) were shown to efficiently abolish Cas12a-mediated gene editing in human cells. Alternatively, synthetic phosphorothioate-modified DNA oligonucleotides (psDNA) can be adopted (Li B. et al., 2018; Marino et al., 2018; Watters et al., 2018; Knott et al., 2019a). AcrVA1 has the property of being a broad-spectrum inhibitor that works efficiently on four Cas12a proteins (AsCas12a, LbCas12a, MbCas12a, and Mb3Cas12a). Since it was already adopted as a component of multi-input multiplicative logic gates in human cells (Kempton et al., 2020), we think that AcrVA1 protein could be used in the future, together with the CRISPR-dCas12a system, for novel design and *in vivo* implementation of digital circuits of variable complexity (more in general, every AcrVA protein can be employed to control gene expression, if associated with CRISPR-dCas12a—based synthetic transcription factors). However, the usage of AcrVA1 still poses some questions that need to be addressed in order to employ this protein into genetic networks. AcrVA1, indeed, inhibits Cas12a-crRNA by cutting the crRNA. It is not known, though, whether AcrVA1 is released from or stays bound to the Cas12a-crRNA complex after carrying out crRNA cleavage. Furthermore, the inhibitory efficiency of AcrVA1 might be dose dependent. This could imply that a sufficiently high amount of crRNA would prevent AcrVA1 from having a significative influence on CRISPR-Cas12a.

AcrVA4 and AcrVA5 are better characterized. Both of them strongly inactivate LbCas12a functioning (Watters et al., 2018; Dong et al., 2019; Knott et al., 2019a). Thus, AcrVA4 and AcrVA5 *in tandem* with LbCas12a are reliable solutions for synthetic gene circuits in eukaryotic cells. In contrast, they are unfunctional on AsCas12a (Watters et al., 2018; Dong et al., 2019; Knott et al., 2019a; Zhang H. et al., 2019). As an alternative, AcrVA4 could be paired to the chimeric As^∗^Cas12a protein, as reported in Knott et al. (2019a). As for AcrVA5, it is worth mentioning that this anti-CRISPR protein functions as an acetyltransferase able to inactivate MbCas12a permanently by covalent modification. This reaction, however, demands the presence of acetyl-CoA (Dong et al., 2019). In principle, AcrVA5 might control MbCas12a-based transcription factor at a relatively low concentration as long as acetyl-CoA is present in the cell in an adequate amount.

Overall, compared to type I, type V-A CRISPR-Cas systems are easier to be converted into transcription factors and used inside gene circuits. Unlike type I, type V-A would demand the expression of a single (nuclease-deficient) Cas protein—dCas12a. Moreover, dCas12a keeps the ability to process its own pre-crRNA array (Zetsche et al., 2015), which could also limit the number of transcription units necessary for circuit construction *in vivo*. However, new computational methods are necessary to estimate, faithfully, the number of off-target sites that appears to be higher when employing a nuclease-deficient Cas—as reported on Cas9 (Wu et al., 2014). Finally, the genetic burden induced by the concomitant expression of Cas proteins and the corresponding anti-CRIPSRs should be assessed carefully, as it was proven to be non-negligible in eukaryotes such as *Saccharomyces cerevisiae* (Li J. et al., 2018).

## Author Contributions

LY and MAM wrote the manuscript. Both authors contributed to the article and approved the submitted version.

## Conflict of Interest

The authors declare that the research was conducted in the absence of any commercial or financial relationships that could be construed as a potential conflict of interest.
